# Reversal of alopecia areata, osteoporosis follow treatment with activation of Tgr5 in mice

**DOI:** 10.1042/BSR20210609

**Published:** 2021-07-20

**Authors:** Xiaohui Zhou, Zhiqiang Guan, Xiao Jin, Jianbin Zhao, Guisheng Chen, Jicun Ding, Yile Ren, Xiaoxiang Zhai, Qiyun Zhou, Zhiyuan Guan

**Affiliations:** 1 Qinghai Provincial People’s Hospital, Xining, Qinghai, P.R. China; 2 Department of Dermatology, The First People's Hospital of Xuzhou, Xuzhou, Jiangsu 221002, P.R. China; 3 Department of Rheumatology and Immunology, The First People’s Hospital of Xuzhou, Xuzhou, Jiangsu 221002, P.R. China; 4 Department of Dermatology, Traditional Chinese Medicine Hospital of Xuzhou Jiangsu 221002, P.R. China; 5 Department of Dermatology, The Seventh People’s Hospital of Shanghai, Shanghai 200137, P.R. China; 6 Department of Orthopedics, The Shanghai Tenth People's Hospital of Tongji University, Shanghai, P.R. China; 7 Department of Orthopedics, Peking University Third Hospital, Beijing, P.R. China

**Keywords:** Alopecia areata, bone, JAK1-STAT3 signaling pathway, Tgr5

## Abstract

Background: Alopecia areata is an autoimmune hair loss disease with infiltration of pro-inflammatory cells into hair follicles. The role of Tgr5 in dermatitis has attracted considerable attention. The present study aimed to investigate the effect of Tgr5 in the development of Alopecia areata.

Methods: The study utilized a comparison control group design with four groups of wild-type group, wild-type+INT777 group, Tgr5^−/−^ group, and Tgr5^−/−^+INT777 group. The mice were treated with INT777 (30 mg/kg/day) or the carrier solution (DMSO) intraperitoneally for 7 weeks, and the back skin was collected and analyzed by histology and immunohistochemistry staining. The lumbar vertebrae 4 has also been analyzed by DXA and Micro-CT.

Results: Tgr5^−/−^ mice displayed the decreasingly significant in hair area and length, skin thickness, and the ratio of anagen and telogen, collagen, and mast cell number and loss the bone mass than WT group. After treating with INT777, the appearance of alopecia areata and bone microstructure has improved. Immunohistochemistry and qPCR analysis showed that activation of Tgr5 can down-regulate the express of JAK1, STAT3, IL-6, TNF-α, and VEGF.

Conclusion: These findings indicate that activation of Tgr5 mediated amelioration of alopecia areata and osteoporosis by down-regulated JAK1-STAT3 signaling pathway.

## Introduction

Alopecia areta (AA) is one of the most common immune diseases characterized by a chronic inflammatory reaction to hair follicles [1,2]. The lifetime incidence of AA is approximately 2% worldwide, and there was no significant difference between the sexes. Alopecia areata is an autoimmune disorder characterized by transient, non-scarring hair loss, and preservation of the hair follicle. Hair loss can take many forms ranging from loss in well-defined patches to diffuse or total hair loss, which can affect all hair-bearing sites [3].

The risk factors of AA are depression and rheumatoid arthritis, systemic lupus erythematosus, type I diabetes, celiac disease, thyroid disease, and other autoimmune conditions [4]. What’s more, the quality of life of a child is often closely related to the severity and age of the disease, rather than the duration of the disease [5]. The prevalence of alopecia is 0.08% and is increasing over time, which is lowered in adults than children [6]. The mechanisms of AA are demonstrated in both innate and adaptive immunity [7]. The pathophysiology of AA is also complex such as T lymphocytes, cytokines, neuropeptides, normal hair cycling, and genetic background all play important roles in the induction and maintenance of the disease [8]. The cause of AA remains unclear. Many kinds of literature have found that alopecia areata is related to innate immunity and acquired immunity. In animal experiments, it is found that IFN-γ-driven immune response, including IFNγ, IFNγ-induced chemokines, and cytotoxic CD8+ T cells are important mechanisms leading to alopecia areata [9]. The cytotoxic CD8+ T-cells, natural killer cells, and cytokines/chemokines like IFNγ, CXCL10, NKG2D+ ligands, viral infections, and vaccinations have the strongest link to the AA [10,11]. The loss of immune privilege in the hair follicle, autoimmune-mediated hair follicle destruction, and the up-regulation of inflammatory pathways may play important role in the progress of AA [12]. And more importantly, the Janus kinase/signal (JAK) transducers and activators of the transcription pathway are up-regulated in alopecia areata but not in normal hair follicles. Alopecia areata is driven by cytotoxic T lymphocytes and is reversed by JAK inhibition [10]. IFN-γ is prominently expressed in lesional skin from patients with AA and is believed to contribute to the collapse of immune privilege through the increased follicular expression of MHC class I and II molecules [13]. Inhibition of JAK-STAT signaling with baricitinib reduces inflammation and Improves cellular homeostasis in progeria cells [14]. The use of oral JAK1/2 inhibitor ruxolitinib can improve the treatment of patients with moderate-to-severe AA [15]. Although there is a certain foundation for the research on the mechanism and treatment of alopecia areata, further research is needed.

G-protein-coupled bile acid receptor, Gpbar1 (TGR5), is a member of the G-protein-coupled receptor (GPCR) superfamily. The Tgr5 is not only the receptor of bile acids but also the receptor for multiple selective synthetic agonists such as 6α-ethyl-23(S)-methyl-cholic acid (INT-777) [16]. In lung cancer cells, Tgr5 receptor activation significantly inhibits STAT3 phosphorylation, thus TNF-α and interleukin, the inflammatory genes were decreased such as TNF-α, IL-6 [17]. The TGR5 gene was overexpressed in A549 cell lines and was significantly decreased in STAT3 activity, which promoted the expression of apoptosis gene Bcl-XL and inhibition of proangiogenic factors [18]. The activation of TGR5 and S1PR2, which regulate itch, keratinocyte proliferation, metabolism, and inflammation, may contribute to WD-exacerbated dermatitis with Th2 and Th17 features. Besides, elevated total BA plays a significant role in inducing dermatitis and cutaneous inflammation [19]. Tgr5 is also an established regulator of bone metabolism which regulates osteoclastogenesis by the AMP‐activated protein kinase (AMPK) signaling pathway [20]. In the present study, several studies have been shown the relationship between Tgr5 and JAK-STAT signaling in cancer. However, the relationship between Tgr5 and JAK-STAT signaling in AA has remained unclear.

Osteoporosis also a common systemic skeletal disorder resulting in bone fragility and increased fracture risk [21]. There is also a link between dermatitis and osteoporosis. Previous studies have found that atopic dermatitis is a risk factor for osteoporosis and bone loss. The use of corticosteroids in clinical studies often occurs at the same time as dermatitis and osteoporosis [22]. In clinical studies, it has also been found that there is a significant relationship between alopecia areata and bone loss [23]. Tgr5 can inhibit osteoclasts and promote osteogenic activity to improve the progress of osteoporosis [20,24]. At the same time, Tgr5 also plays an important role in dermatitis [19,25]. However, there is still a lack of relevant research on the role of Tgr5 in alopecia areata. So is it possible to improve the symptoms of alopecia areata and osteoporosis at the same time by adjusting Tgr5?

The present study thus demonstrates that activation of Tgr5 improves the progress of AA via JAK-STAT and provides a basis for understanding the previously unknown link between Tgr5 and skin diseases.

## Methods

### Animals

The methods of knocking out the Tgr5 gene were performed using the CRISPR/Cas9 system in the C57BL/6J mouse as describe in previous studies [20]. Forty male mice (8 weeks old) were housed under a controlled temperature and a humid environment in a clean and specific pathogen-free room with a healthy breeding colony. The experimental animals were divided into four groups (*n* = 10): WT group, WT+INT777 group, KO group, and KO+INT777 group, and the mice were treated with INT777 (TGR5 agonist, 30 mg/kg/day) or the carrier solution (DMSO) intraperitoneally for 7 weeks and were executed with an overdose of anesthesia (pentobarbital) [26]. The experiment was conducted in the Animal Department of Xuzhou First People’s Hospital.

### Biomarker level in skin homogenates

Circular punch samples of skin (6 mm in diameter) were homogenized using a tissue homogenizer in 1 ml of ice-cold cytokine extraction buffer (0.4 M NaCl, 0.05% Tween 20, 0.5% bovine serum albumin, 0.1 mM phenylmethylsulfonyl fluoride, 10 mM EDTA, and 20 Ki of aprotinin). The homogenates were centrifuged at 13,000×***g*** for 10 min at 4°C, and supernatants were stored at −80°C before analysis. Levels of IL-6, TNF-α, VEGF, cortisol and INF-γ (Meimian Biotechnology, Yancheng, Jiangsu, China) were performed using Enzyme-Linked Immunosorbent Assay (ELISA) Kits.

### Histology

Full‐thickness pieces of mouse skin were fixed in 10% formalin and embedded in paraffin. Sections of 5 μm hair follicles (HF) growth was evaluated according to the following four parameters: skin thickness, hair follicles, the ratio of anagen and telogen, hair cycle score, and bulb diameter [27,28]. The collagen staining was analyzed by Masson’s trichrome stain kit (Sigma–Aldrich, St. Louis, MO). The protocol of Hematoxylin–eosin (H&E) staining and Masson’s trichrome stain has been describing in a previous study [29]. For toluidine blue staining, tissue sections were dewaxed, then stained with toluidine blue for 1–2 min, followed by washing in distilled water. After dehydration in a graded ethanol series, sections were placed in xylene, then mounted and examined by light microscopy [30]. The percentage area of collagen deposition was calculated on the Image-pro Plus 6.0 (Media Cybernetics, Maryland, U.S.A.) by counting the number of clearly stained pixels and comparing it with the total number of pixels [31]. The H&E‐stained, Masson-stained, and Toluidine blue staining slides were observed using a digital slide scanner (NanoZoomer Digital Pathology, Japan).

### Immunohistochemistry staining

In the present study, the following primary and secondary antibodies were used: rabbit anti-pJAK1 (Cell Signaling, Beverly, MA), rabbit anti-pSTAT3 (Cell Signaling), rabbit anti-IL-6 (Cell Signaling). For immunohistochemistry, endogenous peroxidases were quenched in 0.3% H_2_O_2_ in water for 10 min and then blocked with 10% normal goat serum in PBST. For detection of intracellular antigens, tissues were incubated with 100% methanol -20°C and permeabilized in blocking solution with 0.3% Triton X-100. The primary antibody in blocking buffer was applied to tissues for 60 min at room temperature or overnight at 4°C. Sections were washed in PBS and then incubated with goat anti-rabbit (Jackson ImmunoResearch, West Grove, PA) or anti-rat biotinylated IgG (BD Biosciences, San Jose, CA). Staining was detected using Vectastain ABC kit (Vector Labs, Burlingame, CA) and sections were counterstained with hematoxylin. The area of positive area and integral optical density was calculated on the Image-pro Plus 6.0.

### Quantitative real-time polymerase chain reaction

Total RNA from cultured dermal fibroblasts was extracted by the Gene JET RNA purification kit (Thermo Scientific, Waltham, MA) according to the manufacture’s protocol. The rescue experiment was also been carried by analyzing the expression of Tgr5 in the skin of the same alopecia areata, left tibia, and tail on the back from the WT group and the Tgr5-KO group.RNA from mouse skin was extracted by Trizol (Invitrogen Life Technologies). The detailed protocol was described in a previous study [29]. The primers used in the present study such as Tgr5, JAK1, STAT3, TNF-α, IL-6, MMP-1, and MMP-3 have been uploaded in Supplementary Table S1. The relative change in gene expression was analyzed by the 2-ΔΔCT method.

### DXA and Micro-CT analysis

The bone mineral density (BMD) of the lumbar vertebrae (L4) was determined using the soft X-ray apparatus with a small-animal high-resolution collimator (Faxitron® LX-60 Cabinet radiography system, U.S.A.). The specimens were collected in a sample holder with PBS and scanned using Micro-CT(Inveon, Siemens, Erlangen, Germany) at a spatial resolution of 55 kVp, 145 mA, 300 ms integration time, and a voxel resolution of 20 µm obtained from 720 views. A region of interest (ROI) contains 1.5 mm long and 0.5 mm proximal to the growth plate in L4 [32,33]. We analyze trabecular and cortical bone microarchitecture by measuring bone volume (BV) over total volume (TV), trabecular thickness (Tb. Th), trabecular number (Tb. N), and trabecular spacing (Tb. Sp) in the lumbar vertebrae.

### Correlation network analyses

Correlation network analyses were built in R (version 3.5.3, R Project; R Foundation for Statistical Computing, Vienna, Austria) as previously described [34]. The correlation threshold was chosen by *P*<0.05 for global networks. Spearman’s rank correlation was chosen for the network construction because of large differences in variable scales.

### Statistical analysis

All data are presented as the mean ± standard deviation (SD) and *P*<0.05 is a significant difference. Statistical analyses were carried out using GraphPad Prism software 8.02 (La Jolla California U.S.A.). The multiple comparisons testing was performed using one-way ANOVA; all other comparisons were determined by Student’s *t*-test or Mann–Whitney test.

## Result

### The effect of Tgr5 on skin hair and hair follicles in Tgr5- mice

**Figure 1:**
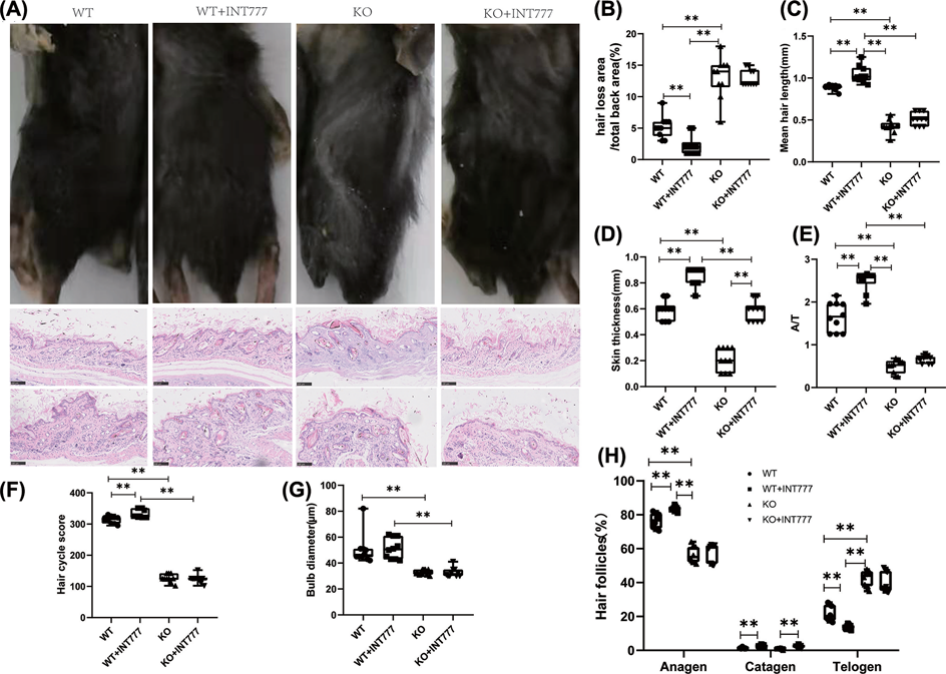
The effect of Tgr5 on skin hair and hair follicles in Tgr5- mice (**A**) Hair loss and hair follicles in HE stain and apparent pictures. (**B**) The ratio of hair loss area and total back area. (**C**) Mean hair length. (**D**) Skin thickness. (**E**) The ratio of anagen and telogen(A/T). (**F**) Hair cycle score. (**G**) Bulb diameter. (**H**) the presence of anagen, catagen, and telogen in hair follicles. Data are expressed as the means ± standard deviation. One-way ANOVA followed by Tukey’s multiple tests has been used; *n* = 10 per group; KO, knockout; WT, wild-type. * *P*<0.05, ** *P*<0.01.

First, we used the rescue experiment to prove the establishment of knockout animals and found that the expression of Tgr5 related genes in the KO group was significantly reduced in the skin, tibia, and tail (Supplementary Table S2). To study AA in detail, we examined hair growth patterns by collecting images, using H&E staining, collagen extracellular matrix deposition. A representative figure has been present in Figure 1A. The ratio of hair loss area and total back area was decreased significantly in WT+INT777 than WT group (*P*<0.01) and increasingly significant in KO group than WT group (*P*<0.01) (Figure 1B). Besides, we also measure the hair length and found that the WT+INT777 group has a longer hair length than the WT group (*P*<0.01), and the KO group was a shorter hair length than the WT group(*P*<0.01) (Figure 1C). Compare with the WT+INT777 group, we found that KO+INT777 also decreased significantly than the WT+INT777 group in hair length(*P*<0.01).

To further investigate the process of AA in Tgr5^−^ hair follicles, we examined skin histology and the detail of the three-stage cycle such as growth (anagen), regression (catagen), and resting phase (telogen). In Figure 1D, the result showed that the WT+INT777 group can increase significantly in skin thickness than the WT group (*P*<0.01) and the KO group also can be decreased significantly in skin thickness than the WT group (*P*<0.01). The growth cycle of hair follicles found that the WT group can increase significantly in anagen and catagen than the WT+INT777 group (*P*<0.01) and decreased significantly in telogen than the WT+INT777 group (*P*<0.01). WT group also decreased significantly in anagen and increasingly significant in telogen than the KO group (*P*<0.01) (Figure 1H). What’s more, the WT group can increase significantly in the ratio of anagen and telogen than the WT+INT777 group (*P*<0.01) and decrease significantly than the KO group (*P*<0.01) (Figure 1E). For the hair cycle score, we found that the WT group can increase significantly than the WT+INT777 group (*P*<0.01) and decreased significantly than the KO group (*P*<0.01). WT+INT777 group also can increase significantly in hair cycle score than KO+INT777 group (*P*<0.01) (Figure 1F). The bulb diameter was decreased significantly in the KO group than the WT group (*P*<0.01), but there is no significant difference between the WT group and KO group in bulb diameter (Figure 1G). The INT777+KO group had no statistical difference in hair loss area, hair length, A/T ratio, hair cycle score, and bulb diameter compared with the KO group, indicating that after knocking out the Tgr5 gene, the agonist of the Tgr5 gene was used to treat no obvious improvement effect in alopecia areata.

### The effect of Tgr5 on collagen and mast cell number in Tgr5-mice

**Figure 2:**
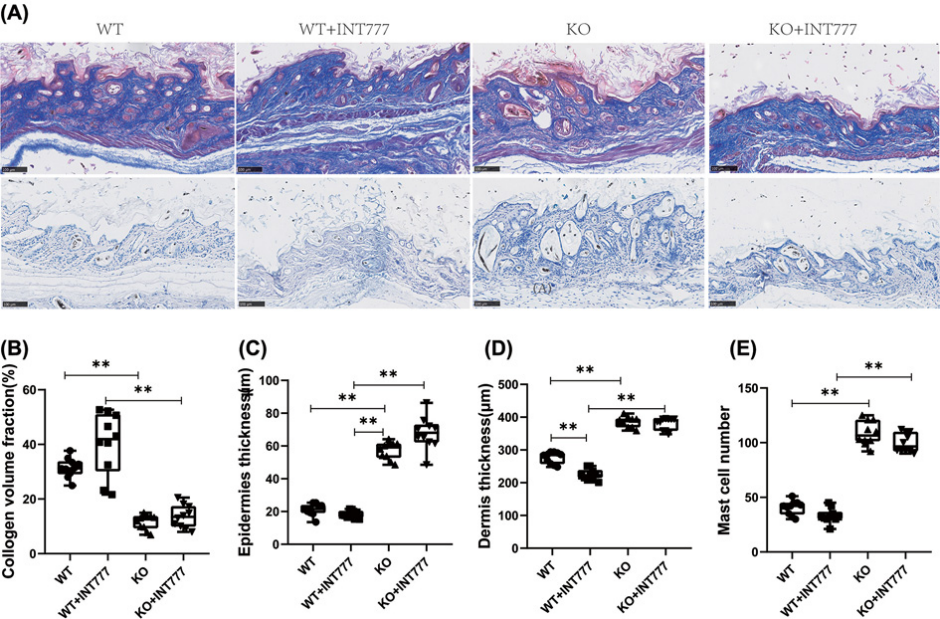
The effect of Tgr5 on collagen and mast cell number in Tgr5-mice (**A**) collagen and mast cell number in Masson stain and Toluidine blue stain. (**B**) Collagen volume fraction. (**C**) Epidemics thickness. (**D**) Dermis thickness. (**E**) Mast cell number. Data are expressed as the means ± standard deviation. One-way ANOVA followed by Tukey’s multiple tests has been used; *n* = 10 per group; * *P*<0.05, ** *P*<0.01. KO, knockout; WT, wild-type

In Figure 2, we used Masson’s stain and toluidine blue stain to investigate the collagen and mast cells in the skin. A representative figure of Masson’s stain and toluidine blue stain has been shown in Figure 2A. The collagen volume fraction was decreased significantly in the KO group than the WT group (*P*<0.01) and increasingly significant in the KO+INT777 group than the WT+INT777 group (*P*<0.01) (Figure 2B). We are also analyzing the epidermis thickness and dermis thickness in AA mice. WT group was decreased significantly than in the KO group (*P*<0.01), and the KO+INT777 group was also increased significantly than WT+INT777 group in epidermis thickness (*P*<0.01) (Figure 2C). WT+INT777 group was decreased significantly in dermis thickness than WT group (*P*<0.01) and increasingly significant in the KO group than the WT group (*P*<0.01) (Figure 2D). For the mast cell number, we found that the KO group can increase significantly in mast cell number than the WT group (*P*<0.01), and the KO+INT777 group was also increased significantly than WT+INT777 group (*P*<0.01) (Figure 2E).

### The effect of Tgr5 on the JAK1-STAT3 expression in Tgr5- mice

**Figure 3:**
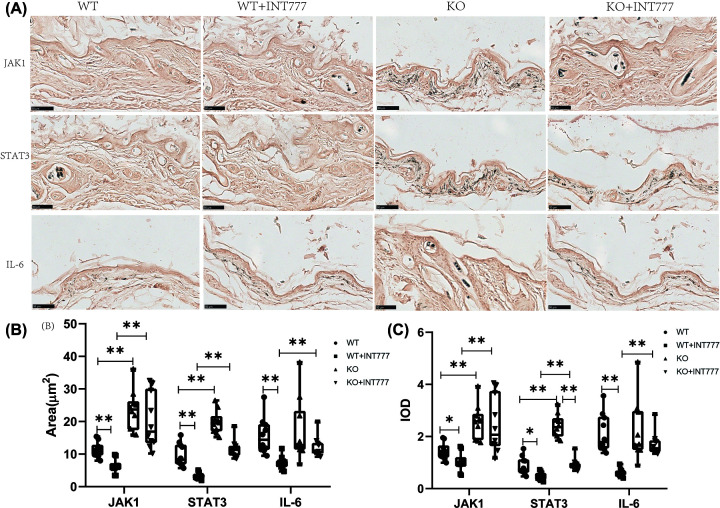
The effect of Tgr5 on Immunohistochemical results in Tgr5- mice (**A**) Immunohistochemical representation of JAK1,STAT3, and IL-6. (**B**) Positive cell area. (**C**) IOD. Data are expressed as the means ± standard deviation. One-way ANOVA followed by Tukey’s multiple tests has been used; *n* = 10 per group; * *P*<0.05, ** *P*<0.01. KO, knockout; WT, wild-type.

In Figure 3, we investigated the JAK1, STAT3, IL-6 expression on IHC and the representative figure also has been shown in Figure 3. The result showed that the positive cell area of the WT+INT777 group was decreased significantly in JAK1, STAT3, IL-6 than the WT group (*P*<0.01) and the KO group also increased significantly in JAK1, STAT3 than WT group (*P*<0.01). KO+INT777 group was also increased significantly in JAK1, STAT3, IL-6 than WT+INT777 group (*P*<0.01) (Figure 3B). For IOD, we also found that the KO group also increased significantly the expression of JAK1 (*P*<0.01), STAT3 (*P*<0.01) than WT group, and the WT+INT777 group was decreased significantly the expression of JAK1 (*P*<0.05), STAT3 (*P*<0.05), IL-6 (*P*<0.01) in WT group (Figure 3C).

### The effect of Tgr5 on the biomarker and mRNA in Tgr5- mice

**Figure 4:**
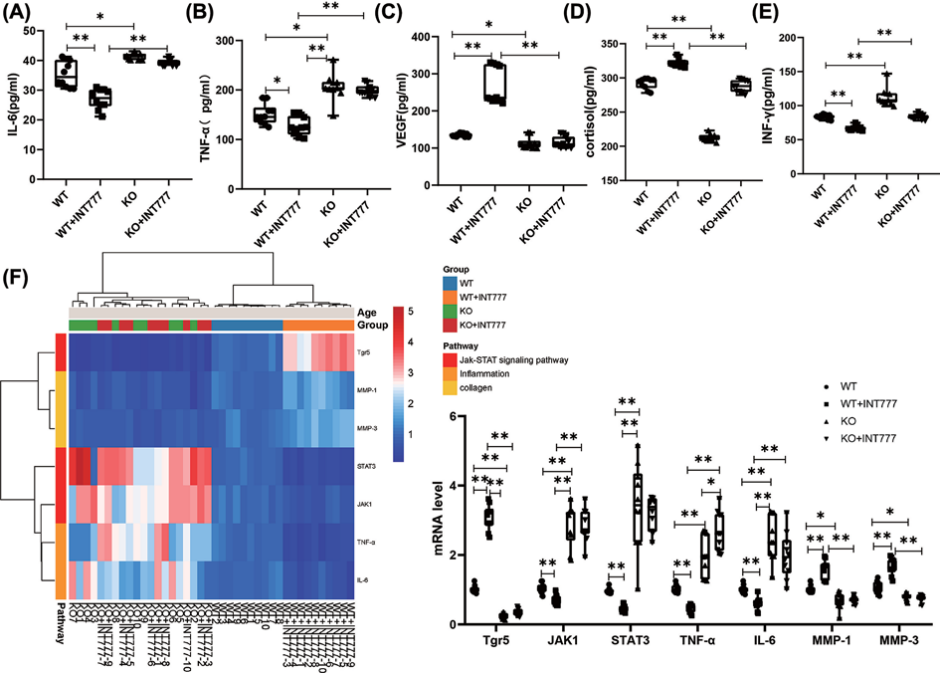
The effect of Tgr5 on the biomarker and mRNA in Tgr5- mice (**A**) IL-6. (**B**) TNF-α. (**C**) VEGF. (**D**) Cortisol. (**E**) INF-γ. (**F**) Heat map and histogram of mRNA expression in the skin. Data are expressed as the means ± standard deviation. One-way ANOVA followed by Tukey’s multiple tests has been used; *n* = 10 per group; IL-6, interleukin-6; INF-γ, interferon-γ; JAK1, Janus kinase1; KO, knockout; MMP-1, matrix metalloproteinase-1; STAT3, signal transduction and activator of transcription; Tgr5, G-protein-coupled bile acid receptor-5; TNF-α, tumor necrosis factor-α; * *P*<0.05, ** *P*<0.01. VEGF, vascular endothelial growth factor; WT, wild-type.

The biomarker such as IL-6, TNF-α, VEGF, cortisol, and INF-γ has been tested in our studies. WT group also can decrease significant in IL-6 (*P*<0.05), TNF-α (*P*<0.05), VEGF (*P*<0.05), cortisol (*P*<0.01), and INF-γ (*P*<0.05) than KO group and increasingly significant in IL-6 (*P*<0.01), TNF-α (*P*<0.05), VEGF (*P*<0.01), cortisol (*P*<0.01), and INF-γ (*P*<0.05) than WT+INT777 group (Figure 4A–E). To investigate the expression of mRNA level in the skin, the heatmap has been uploaded in Figure 4F. The WT group can decrease significantly in the expression of mRNA level of Tgr5 (*P*<0.01), MMP-1 (*P*<0.01), MMP-3 (*P*<0.01), and increasingly significant in the expression of mRNA level of JAK1 (*P*<0.01), STAT3 (*P*<0.01), TNF-α (*P*<0.01), IL-6 (*P*<0.01) than WT+INT777 group. KO group was also decreased significantly in the expression of mRNA level of Tgr5 (*P*<0.01), MMP-1 (*P*<0.05), MMP-3 (*P*<0.05) and increasingly significant in the expression of mRNA level of JAK1 (*P*<0.01), STAT3 (*P*<0.01), TNF-α (*P*<0.01), IL-6 (*P*<0.01) than WT group (Figure 4D).

### The effect of Tgr5 on bone microstructure in Tgr5- mice

**Figure 5:**
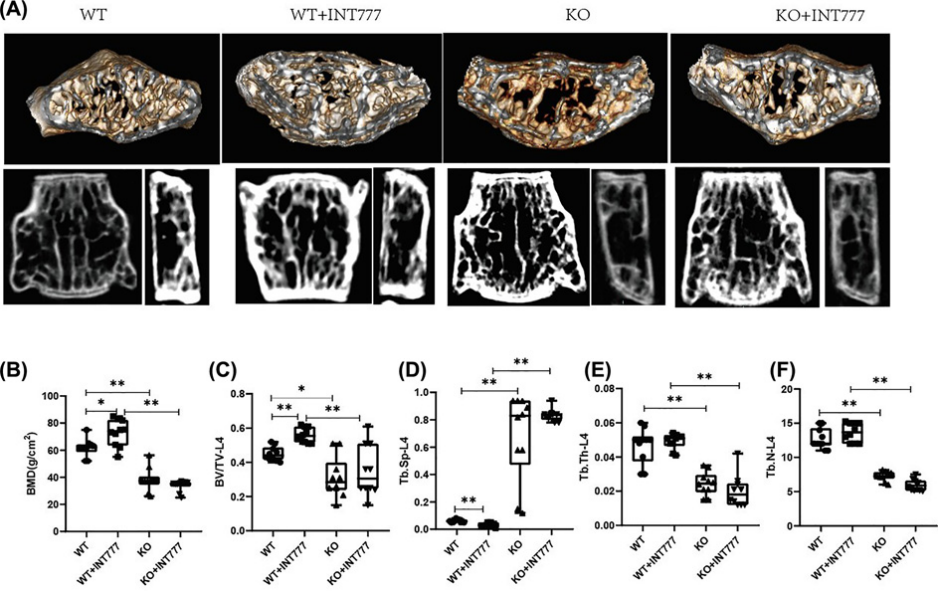
The effect of Tgr5 on bone microstructure in Tgr5- mice (**A**) Representative picture of bone microstructure in Tgr5- mice. (**B**) BMD. (**C**) BV/TV. (**D**) Tb. Sp. (**E**) Tb. Th. (**F**) Tb.N. Data have expressed as the means ± standard deviation. One-way ANOVA followed by Tukey’s multiple tests has been used; *n* = 10 per group; BMD, bone mineral density; BV/TV, bone volume over total volume; * *P*<0.05, ** *P*<0.01. KO, knockout; Tb.Th, trabecular thickness; Tb. N, trabecular number; Tb.Sp, trabecular spacing; WT, wild-type.

To investigate the effect of Tgr5 on bone microstructure, we used the DXA and Micro-CT to analyze the development of bone microstructure. The representative figure also has been shown in Figure 5A. WT+INT777 group was increased significantly in BMD-L4 (*P*<0.05), BV/TV-L4 (*P*<0.01), and decreased significantly in Tb.Sp-L4 (*P*<0.01) than WT group. KO group was decreased significantly in DXA (*P*<0.01), BV/TV-L4 (*P*<0.05), Tb.Th-L4 (*P*<0.01), Tb.N-L4 (*P*<0.01), and increasingly significant in Tb.Sp-L4 (*P*<0.01) than WT group. Besides, KO+INT777 was decreased significantly in DXA (*P*<0.01), BV/TV-L4 (*P*<0.01), Tb.Th-L4 (*P*<0.01), Tb.N-L4 (*P*<0.01), and increasingly significant in Tb.Sp-L4 (*P*<0.01) than WT+INT777 group (Figure 5B–D).

### Correlation of Tgr5 and AA, osteoporosis in Tgr5- mice

To evaluate the possible correlation between Tgr5 and AA, osteoporosis in mice, the correlation network analysis was built-in Supplementary Figure S1A. the result found that the specific pairs of correlated with variables were almost entirely different between KO groups and KO+INT777 groups, WT group and KO group, KO group and WT+INT777 group, WT group, and WT+INT777 groups, with only 2,2,4 4 common correlated pairs (Supplementary Figure S1B). To better understand potential links to Tgr5 and AA, Osteoporosis, we focused on the primary network of skin indicators for example skin thickness. Skin thickness was positively correlated with Tgr5 mRNA, A/T, hair length, cortisol but negatively correlated with IL-6, IL-6 mRNA, STAT3-IOD, INF-γ, dermis thickness, Telogen (Supplementary Figure S1C and D). The detailed correlation networks of the whole and each group also has been analyzed in Supplement Figures S2–6.

## Discussion

TGR5 has been analyzed in previous studies by regulating the JAK1/STAT3 signaling pathway [17,18]. We tested the role of Tgr5 in the affected skin and conducted a series of experiments. First, we stained the skin samples for Tgr5- and Tgr5+ mice with or not Tgr5 agonists. Pathological findings that Tgr5 play important role in the improvement of skin thickness, hair length, hair loss, and hair follicles in HE stain. What’s more, the collagen and mast cell number also can improve by activation of Tgr5. Secondly, we further analyzed the expressions of the JAK1-STAT3 signaling pathway and other related proteins and mRNA in skin tissues. We found that TGR5 can down-regulate the expression of JAK1 and STAT factors, and then improve the expression of inflammation-related genes in skin tissue. Finally, We further verified the relationship between the expression of related genes and AA by network correlation analysis.

The role of TGR5 in skin-related diseases has attracted more and more attention. Jena et al. found that activation of TGR5 which regulates itch, keratinocyte proliferation, metabolism, and inflammation, may contribute to Western diet-exacerbated dermatitis [19]. Chang et al. also found that the protective effects of TGR5 against ultraviolet B irradiation in epidermal stem cells [35]. The Tgr5 also play important role in skin itch and analgesia. Alemi et al. found that bile acids activate TGR5 on sensory nerves, stimulating the release of neuropeptides in the spinal cord that transmit itch and analgesia [25]. Cipriani et al. also found that the itching response to intradermal injection of GPBAR1 agonists desensitizes rapidly and is deactivated in models of cholestasis, which explains the lack of correlation between bile acids levels and itching severity in cholestatic syndromes [36]. In our study, we found that TGR5 is closely related to the JAK-STAT signaling pathway. AA is determined by the loss of the immune-privileged status of the hair follicles, which are then attacked by autoreactive CD8+ T cells and by NK T cells. The effect of tofacitinib on the JAK/STAT pathway in AA is evidenced by the down-regulation of pSTAT3 in hair follicles during treatment. Remarkably, hair follicles have developed different mechanisms to maintain their privileged immune status [37]. A recent study of normal murine cycling hair demonstrated that JAK signaling induces a telogen block, preventing the hair from entering an anagen phase and that JAK inhibitors induce anagen hair growth similar to a hedgehog pathway agonist [38]. Solimani et al. found that experimental AA mouse models can be increasingly significant in levels of phosphorylated STAT proteins, specifically STAT1, which are activated downstream the signals from IFN-γ, IL-2, and IL-15 [10]. Janus kinase (JAK) inhibitors could be considered effective and well-tolerated treatments for extensive AA [39]. Julian et al. found that 9 of 12 patients (75%) treated with ruxolitinib showed significant scalp hair regrowth and improvement of AA [15]. What’s more, atopic dermatitis is characterized by an inflammatory microenvironment which activates cytokine receptors coupled to the Jak-Stat signaling pathway [40]. Kim et al. investigate that JAK inhibitors such as ruxolitinib modulated and reverted the interferon-induced inflammatory changes by blocking the JAK-STAT pathway in hDPCs under an AA-like environment [41]. JAK-STAT pathway as an emerging target for topical treatment of inflammatory skin diseases [42].

Besides, the hair follicle undergoes pronounced cyclic expansion and regression, leading to rapidly changing demands for its vascular support. Angiogenic and/or lymphangiogenic factors may represent therapeutic targets in inflammatory skin disorders, such as atopic dermatitis. Aanhold et al. found that the vascular endothelial growth factor inhibitor ameliorates atopic dermatitis in APOC1 transgenic mice [43]. Ozeki et al. found that VEGF is a marker of angiogenesis, stimulating hair growth by facilitating the supply of nutrients to the hair follicle and increasing follicular diameter [44]. Tgr5 also plays important role in bone metabolism. Wang et al. found that activation of TGR5 promotes osteoblastic cell differentiation and mineralization [24]. Li et al. also found that Tgr5 regulates osteoclastogenesis by the AMP-activated protein kinase (AMPK) signaling pathway, which is a central metabolic pathway involved in the pathophysiology of aging and age-related diseases [20]. Based on our finding of a correlation between Tgr5, AA, and osteoporosis, we used network correlation analysis to further verify the relevant results, but due to the limited sample size, further verification of the results is needed in the future.

The relationship between osteoporosis and dermatitis has also attracted people’s attention. A clinical study in the United States found that adults with dermatitis have a significantly increased risk of osteoporosis and bone loss [45]. In a study in Taiwan, China, it was found that the risk of osteoporosis in adults with dermatitis increased by four times, especially in elderly women, or in patients with depression and corticosteroids [46]. Therefore, some studies suggest that patients with more than moderate dermatitis should pay attention to the screening of osteoporosis and bone loss [22,47]. Alopecia areata is another typical skin-related disease. Alopecia areata, especially patients with long-term oral steroids, have a significantly increased risk of osteoporosis [23]. However, the relevant mechanism is currently unclear. In the present study, the intervention of Tgr5 not only improved the symptoms of alopecia areata but also improved bone metabolism disorders, which has certain positive significance for the future treatment of patients with alopecia areata accompanied by osteoporosis.

## Conclusion

In summary, activation of Tgr5 has improved the development of AA and bone microstructure which is related to the JAK1-STAT3 signaling pathway, inflammatory state, and the angiogenic factor. The mechanism of Tgr5 mediated amelioration of alopecia areata offers new insights into the treatment of alopecia areata, and it suggests novel therapeutic approaches for the treatment of alopecia areata and osteoporosis.

## Supplementary Material

Supplementary Figures S1-S6 and Tables S1-S2

## Data Availability

All supporting data are included within the main article.

## Abbreviations

AA: alopecia areta
AMPK: AMP-activated protein kinase
BMD: bone mineral density
GPCR: G-protein-coupled receptor
H&E: Hematoxylin–eosin
JAK: Janus kinase
TNF-α: tumor necrosis factor-α
VEGF: vascular endothelial growth factor
