# Correction: Reversal of alopecia areata, osteoporosis follow treatment with activation of Tgr5 in mice

**DOI:** 10.1042/BSR-2021-0609_COR

**Published:** 2023-05-23

**Keywords:** Alopecia areata, bone, JAK1-STAT3 signaling pathway, Tgr5

The authors of the original article “Reversal of Alopecia Areata, Osteoporosis follow treatment with Activation of Tgr5 in mice” (*Biosci Rep*. 2021 41(7): BSR20210609 doi: 10.1042/BSR20210609) would like to correct Figure 3. The authors stated that a duplication occurred between the IL-6 immunohistochemical pictures in the WT+INT777 group and KO+INT777 group of Figure 3, due to errors during the import process from Adobe Illustrator. The requested correction has been assessed and agreed by the Editorial Board. The authors declare that these corrections do not change the results or conclusions of their paper. The corrected version of Figure 3 is presented here.

**Figure 3:**
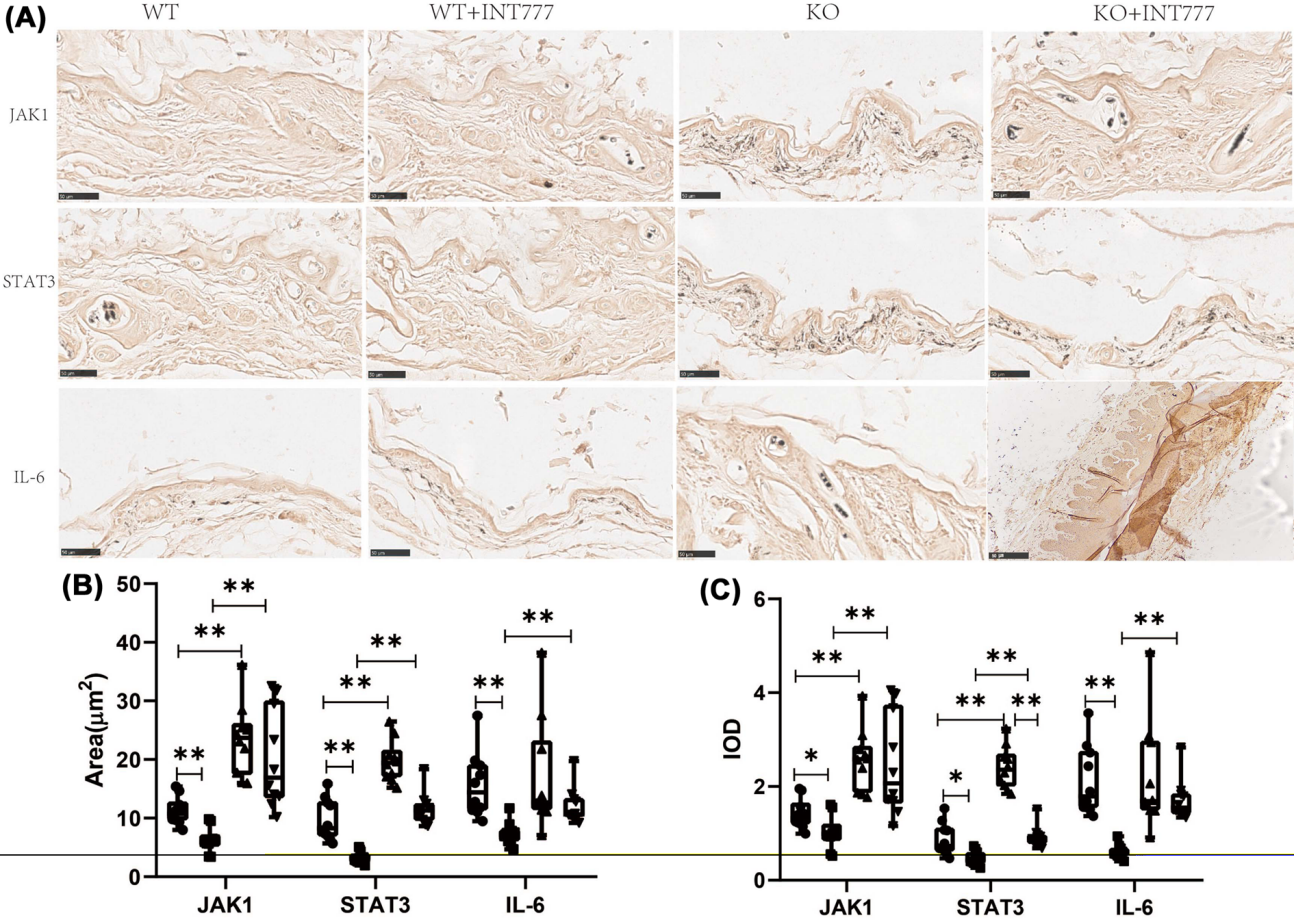
The effect of Tgr5 on Immunohistochemical results in Tgr5- mice **(A)** Immunohistochemical representation of JAK1,STAT3, and IL-6. **(B)** Positive cell area. **(C)** IOD. Data are expressed as the means ± standard deviation. One-way ANOVA followed by Tukey’s multiple tests has been used; n = 10 per group; * P<0.05, ** P<0.01. KO, knockout; WT, wild-type.

