# Supplementary material for: Reversal of alopecia areata, osteoporosis follow treatment with activation of Tgr5 in mice
Source: Biosci Rep. 2021 Jul 20;41(7):BSR20210609. doi: 10.1042/BSR20210609 (PMC8292761; doi:10.1042/BSR20210609)
Supplement: Supplementary Figures S1-S6 and Tables S1-S2 [file BSR-2021-0609_supp.pdf]

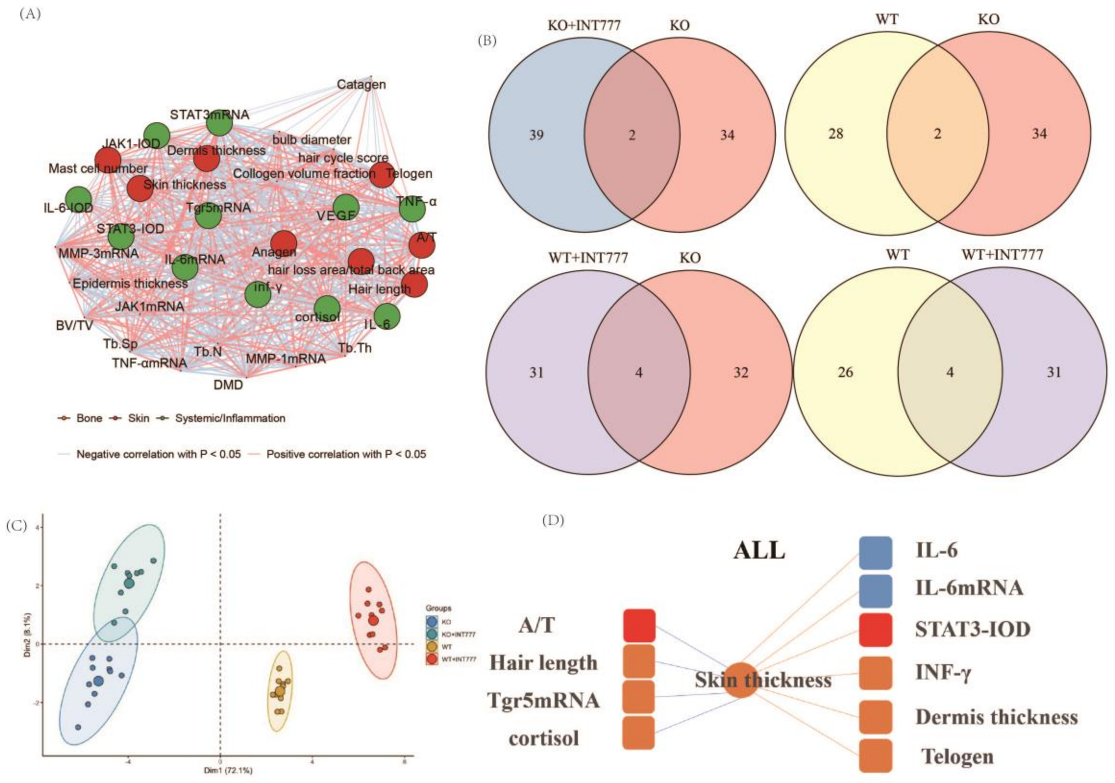

Supplement Figure 1 Correlation of Tgr5 and AA, Osteoporosis in Tgr5- mice. **(A)** Lines show correlations based on Spearman's rank analysis with $p < 0.05$, with line thickness proportional to the correlation strength. **(B)** Venn diagrams comparing unique and shared Correlations between WT group, WT+INT777 group, KO group, KO+INT777 group. **(C)**Principal coordinate analysis. **(D)** Primary "first neighbor" network of skin thickness. Data are expressed as the means ± standard deviation. One-way ANOVA followed by Tukey's multiple tests has been used. n=10 per group. WT: wild type, KO: Knock Out. IOD: integrated Optical Density.

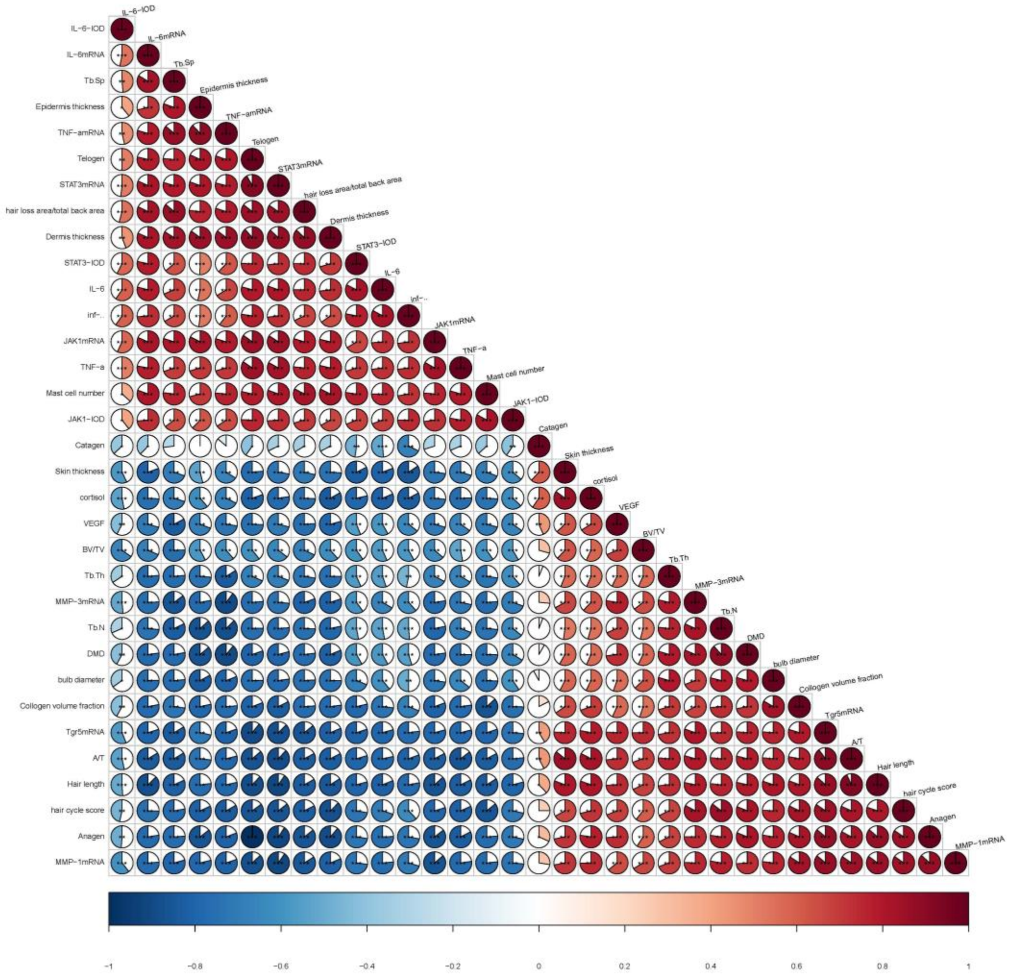

Supplement Figure 2 The detailed correlation networks of All groups.* *P*<0.05,***P*<0.01. IL-6, Interleukin-6. IOD, integrated optical density. TNF-α, tumor necrosis factor-α.STAT3, Signal transducer, and activator of transcription 3. VEGF, vascular endothelial growth factor. INF-γ, interferon-γ.Tgr5: G-protein-coupled bile acid receptor-5. JAK1, Janus kinase1.MMP-1: matrix metalloproteinase-1. MMP-3: matrix metalloproteinase-3.

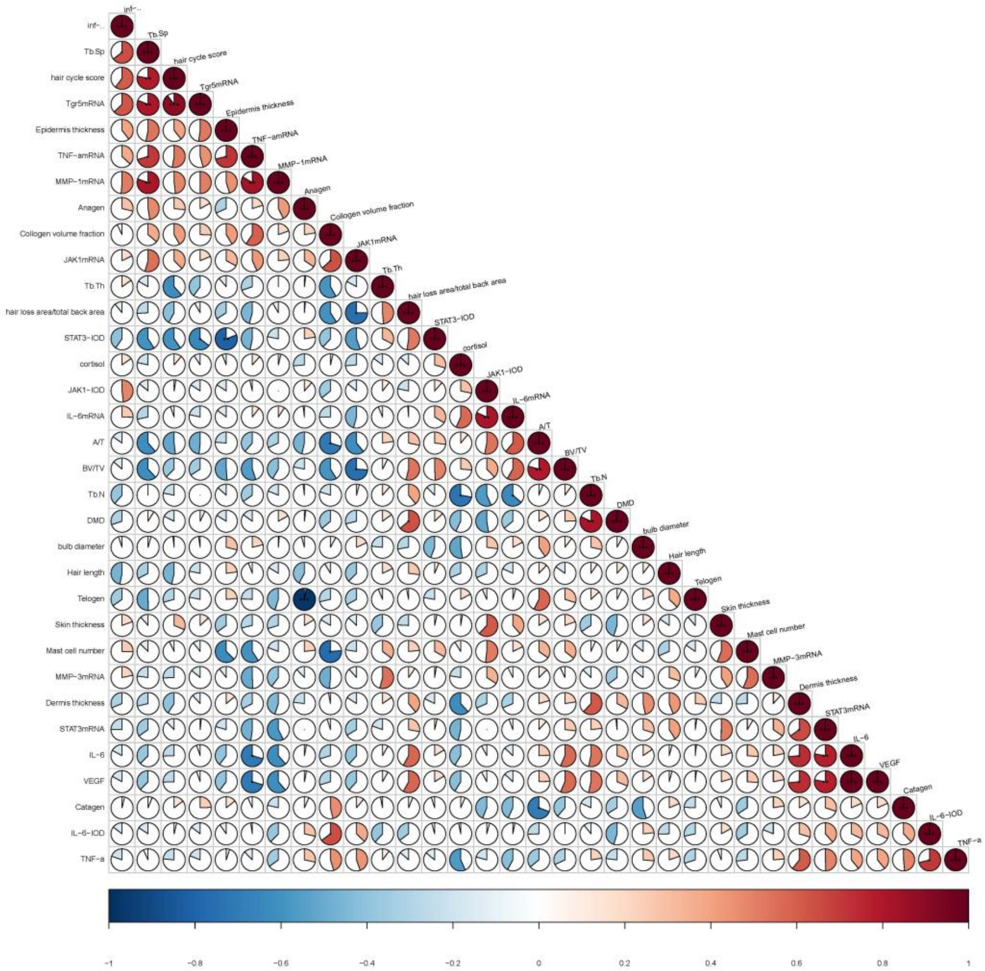

Supplement Figure 3 The detailed correlation networks of the WT group. n=10 per group. * *P*<0.05,***P*<0.01. IL-6, Interleukin-6. IOD, integrated optical density. TNF-α, tumor necrosis factor-α.STAT3, Signal transducer, and activator of transcription 3. VEGF, vascular endothelial growth factor. INF-γ, interferon-γ.Tgr5: G-protein-coupled bile acid receptor-5. JAK1, Janus kinase1.MMP-1: matrix metalloproteinase-1. MMP-3: matrix metalloproteinase-3.

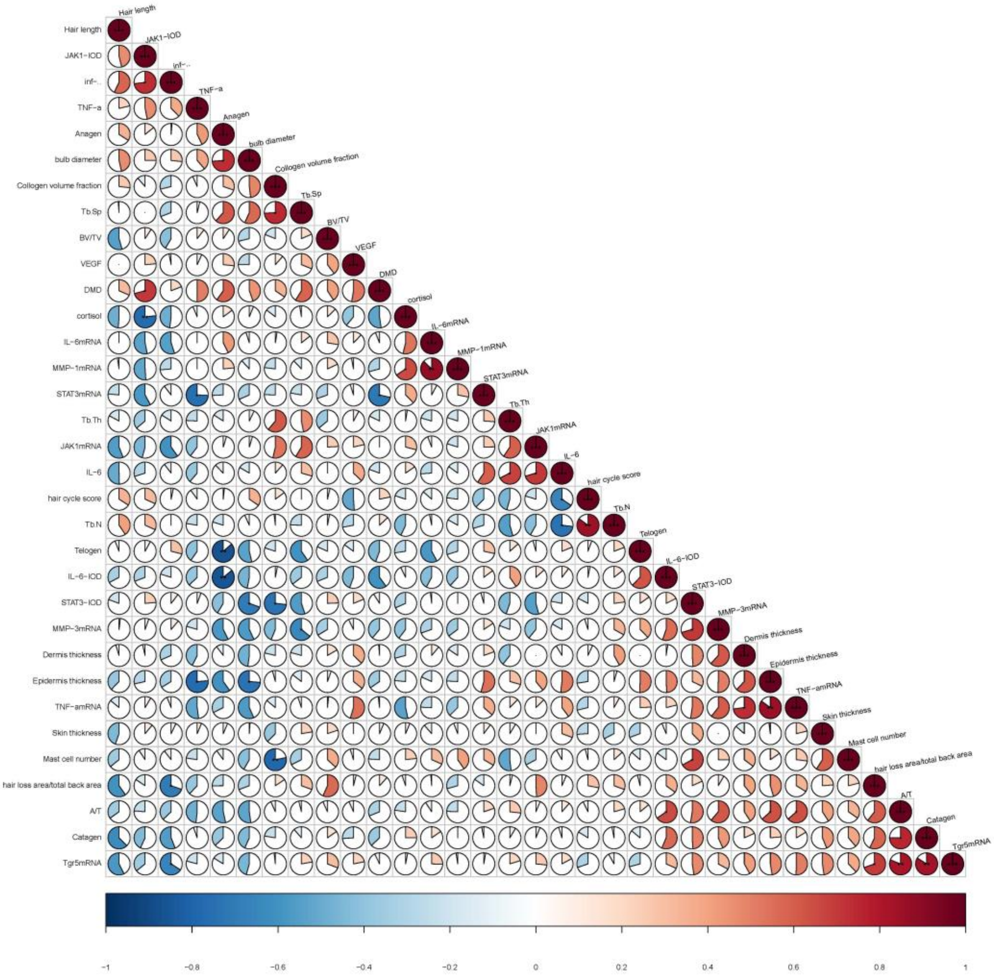

Supplement Figure 4 The detailed correlation networks of WT+INT777 group. * P<0.05,** P<0.01. IL-6, Interleukin-6. IOD integrated optical density. TNF-α, tumor necrosis factor-α.STAT3, Signal transducer, and activator of transcription 3. VEGF, vascular endothelial growth factor. INF-γ, interferon-γ.Tgr5: G-protein-coupled bile acid receptor-5. JAK1, Janus kinase1.MMP-1: matrix metalloproteinase-1. MMP-3: matrix metalloproteinase-3.

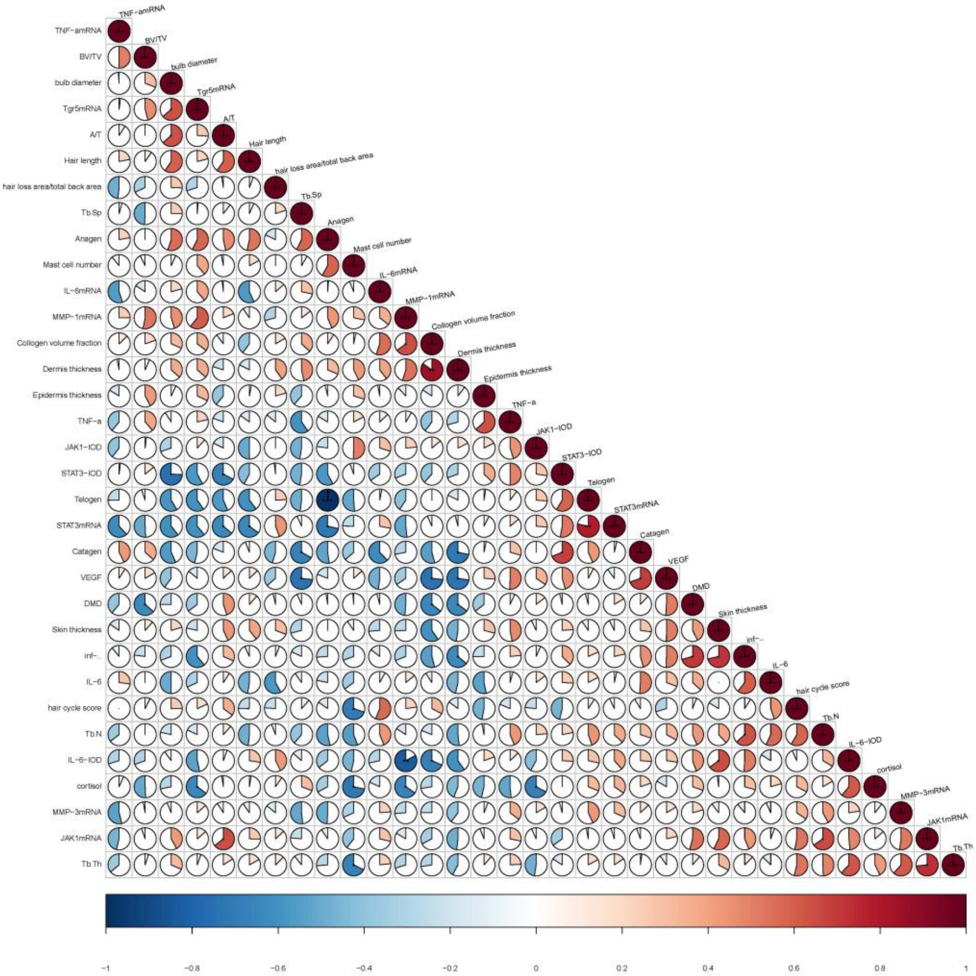

Supplement Figure 5 The detailed correlation networks of the KO group. * *P*<0.05,** *P*<0.01. IL-6, Interleukin-6. IOD integrated optical density. TNF-α, tumor necrosis factor-α.STAT3, Signal transducer, and activator of transcription 3. VEGF, vascular endothelial growth factor. INF-γ, interferon-γ.Tgr5: G-protein-coupled bile acid receptor-5. JAK1, Janus kinase1.MMP-1: matrix metalloproteinase-1. MMP-3: matrix metalloproteinase-3.

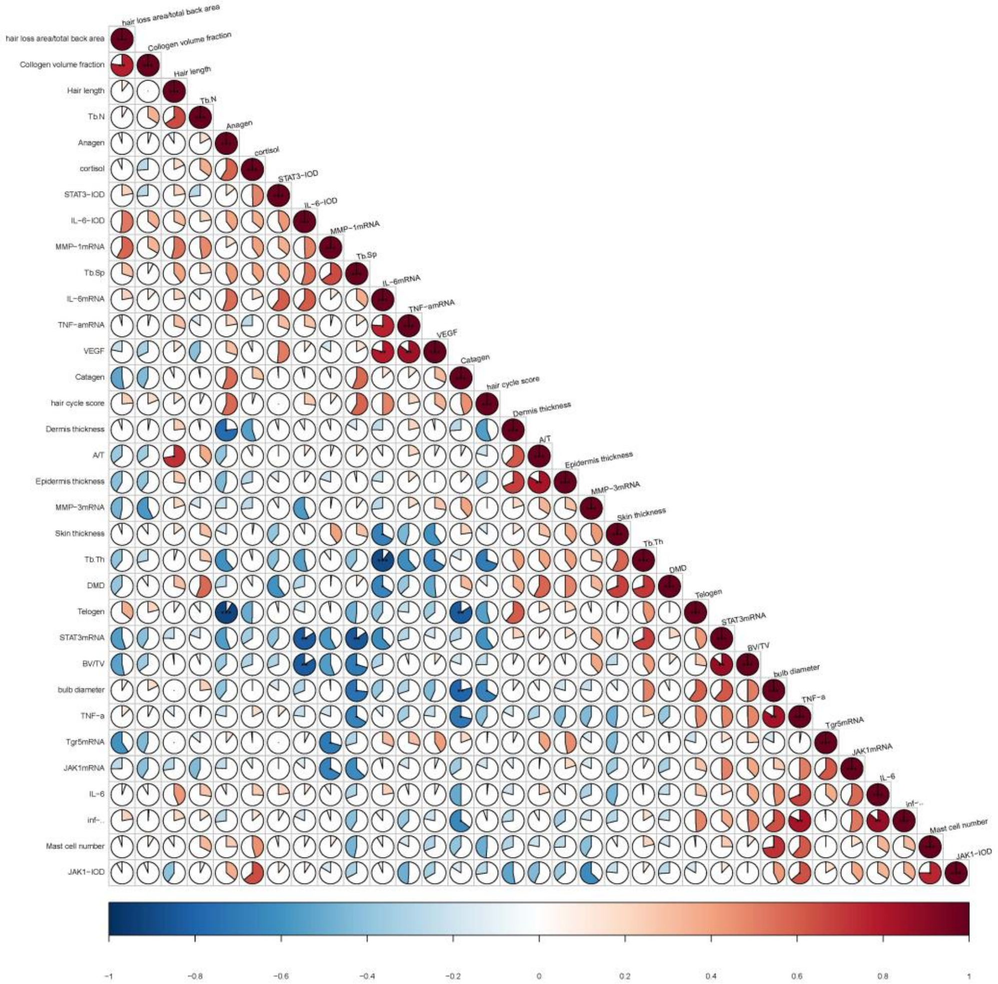

Supplement Figure 6 The detailed correlation networks of the KO+INT777 group. * *P*<0.05,***P*<0.01. IL-6, Interleukin-6. IOD integrated optical density. TNF-α, tumor necrosis factor-α.STAT3, Signal transducer, and activator of transcription 3. VEGF, vascular endothelial growth factor. INF-γ, interferon-γ.Tgr5: G-protein-coupled bile acid receptor-5. JAK1, Janus kinase1.MMP-1: matrix metalloproteinase-1. MMP-3: matrix metalloproteinase-3.

Supplement Table 1: Primer Sequences used for RT-qPCR		
	Forward primer sequences	Reverse primer sequences
GAPDH	5’-AACTTTGGCATTGTGGAAGG-3’	5′ACACATTGGGGGTAGGAACA-3’
JAK1	5’- - TGCACCGACTTTGACAACATT-3’	5′TGAGGTGGTTCATGAGGTCTC-3’
STAT3	5’-TGGGCATCAATCCTGTGGTAT -3’	5′- - TAGTTCACACCAGGCCCTAAG-3’
MMP-1	5’CAGAGATGAAGTCCGGTTTTTC-3’	5′-GGGGTATCCGTGTAGCACAT-3’
TNF-a	5’- AGGCTGCCCCGACTACGT-3’	5’- GACTTTCTCCTGGTATGAGATAG CAA-3’
IL-6	5’-GGCGGATCGGATGTTGTGAT-3’	5′-GGACCCCAGACAATCGGTTG-3’
MMP-3	5’-GTCCTCCACAGACTTGTCCC-3’	5′-AGGACATCAGGGGATGCTGT-3’
TGR5	5'-CCCAACUUCUCCUUCCUCUTT-3'	3'-AGAGGAAGGAGAAGUUGGGTT-5'

Supplement Table 2: the Tgr5 mRNA level in the rescue experiment

	WT group	KO group
Left tibia	1.156±0.098	0.141±0.051**
Tail	1.026±0.105	0.122±0.032*
Skin	1.119±0.163	0.195±0.072**

* Compare with WT group, * P<0.05, ** P<0.01
